# Effects of Clipping an Invasive Plant Species on the Growth of Planted Plants of Two Co-Occurring Species in a Greenhouse Study

**DOI:** 10.3390/biology12101282

**Published:** 2023-09-26

**Authors:** Xiaoqi Ye, Jinliu Meng, Ruixiang Ma, Ming Wu

**Affiliations:** Research Station of Hangzhou Bay Wetland Ecosystems, Institute of Subtropical Forestry, Chinese Academy of Forestry, Hangzhou 311400, China; mengxqi@caf.ac.cn (X.Y.); mengjinliu0542@sina.com (J.M.); maruixiang0724@163.com (R.M.)

**Keywords:** *Solidago canadensis*, priority effects, competition, exotic plant invasion, co-occurring species

## Abstract

**Simple Summary:**

Invasive exotic plant species are threats to native flora and other taxa. No effective and environmentally friendly approaches are available for controlling *Solidago canadensis*, an aggressively invasive plant species in China and Europe. We determined that in addition to the traditional measure of clipping, planting plants of two co-occurring and competitive species can further suppress regrowth of clipped *S. canadensis* plants and both the aboveground and belowground part of *S. canadensis* contributed to its suppression effects on planted co-occurring species. These results suggest that incorporation of utilizing biotic resistance from some highly competitive plant species and overcoming belowground priority effects of invasive species into a comprehensive management plan will substantially increase the efficiency of invasive plant control.

**Abstract:**

The restoration of native plants in invaded habitats is constrained with the presence of highly competitive exotic species. Aboveground removal, such as clipping or mowing, of invasive plants is required for successful restoration. The effects of clipping an invasive plant species, *Solidago canadensis*, grown at five densities (1–5 plants per pot), and planting two co-occurring and competitive species, *Sesbania cannabina* and *Imperata cylindrica*, on the growth of both the invasive species and the co-occurring species were investigated in a greenhouse experiment. The established *S. canadensis* suppressed the growth of planted seedlings with 47.8–94.4% reduction in biomass, with stronger effects at higher densities; clipping significantly reduced 97.5–97.4% of biomass of *S. canadensis* and ameliorated the suppression effects (with only 8.7–52.7% reduction in biomass of the co-occurring plants), irrespective of density. Both the aboveground and belowground part of *S. canadensis* contributed to its suppression effects on planted co-occurring species. Seed sowing of co-occurring species reduced the belowground growth, but not the underground growth of *S. canadensis*. *S. cannabina* appeared to be more effective at reducing the growth of *S. canadensis* than *I. cylindrica*. Therefore, clipping together with planting competitive species that can overcome the belowground priority effects of *S. canadensis* could be a promising strategy for controlling *S. canadensis* invasion and restoring native plant communities.

## 1. Introduction

Invasive exotic species are non-native species introduced outside their native range either naturally or anthropogenically, perform luxuriant growth [1], and pose serious impacts on the native species and ecosystems [2,3,4]. The global spread and increasing abundance of some invasive alien species outside their natural range have caused biodiversity declines, agricultural yield reductions, and ecosystem service impairments [5,6,7]. These invasive exotics perform better in comparison with co-occurring native species, which is considered a reason behind the successful establishment of these invaders [8,9]. In the invaded sites, these invaders cause severe impacts on the biodiversity and harm the flora and have become a major concern in natural conservation [10,11,12]. A meta-analysis indicated that invasive alien plant species caused 50.7% reduction of native plant species diversity [6]. Efficient approaches are urgently required to reduce the negative effects of these species and restore native ecosystems [7,13]. The suppression effects of established invasive exotics on native plants are a major constraint to successful restoration, owing to their stronger priority effects, higher competitive abilities, and asymmetric plant size differences [13,14,15]. Therefore, reducing the priority effects of the invasive exotics and enhancing growth and competitive capacity of natives are the key steps of a successful management [13,16,17].

Intervention of growth of invasive exotics (mostly that of the aboveground part) with mowing or clipping, prescribed fire, and spraying herbicides are common approaches to control invasive exotics [18,19,20]. Aboveground clipping or mowing can weaken priority effects by reducing their growth vigor and competitive strength with native species [21,22,23,24,25]. For example, harvesting can reduce the dominance of invasive *Typha* species and promote native species diversity [26]; long-term repeated mowing converted a site dominated with invasive *Arrhenatherum elatius* to a prairie dominated with native grasses [21]; turf stripping together with native seed addition can achieve a 75% reduction of the invasive *Solidago* cover [25]. However, priority effects of some established invasive exotic species can persist if they are exposed to short-term aboveground disturbance due to the earlier belowground biomass formation and soil space occupancy [27]. For instance, Li et al. (2015) found that successful replacement of invasive *Ipomoea cairica* with native species could only occur when both the aboveground and belowground parts of the invasive plants were removed [24]. Furthermore, some invasive exotics have a high and fast regeneration capacity after aboveground disturbance; consequently, they may re-establish a highly competitive capacity against native species [28,29]. Van Kleunen et al. (2004) showed that the partial removal of plant tissues led to temporary growth reductions in invasive *Solidago canadensis*; however, this was compensated with increased growth [30]. Huang et al. (2018) found that some invasive exotics have a higher regrowth capacity and maintain their performance advantages over native species after clipping [29]. These studies indicate that there are uncertainties regarding whether invasive plant species can be controlled successfully using only aboveground disturbances.

In the last decade, applying ecological principles to management of invasive species and restoration of native plant communities has drawn increasing attention [13,16,17], as physical removal or herbicide application failed to economically and effectively control re-invasion and caused secondary environmental problems [31,32,33]. Biotic resistance from native vegetation is one of the key determinants of invasion success [16,17,34,35]. Establishing communities with competitive native species can support further suppression of invasive exotics in addition to traditional approaches [16,17,31]. Some greenhouse experiments have found that native species may help suppress exotic plants and favor the growth of desired native perennials [36,37,38]; however, these studies did not consider the priority effects of invasive exotic species [13,39,40]. Due to the presence of established invasive plants, native grass seeding alone could not inhibit the growth of invasive *Cirsium arvense* [41]. This suggests that whether planting native plants can significantly suppress the growth of invasive exotics may depend on the priority effects of the latter. Furthermore, competition between plants can be significantly altered using density, and higher densities intensify competition for resources between invasive and native species [42,43]. Invasive plant species gain increasing density as invasion proceeds, and the successful establishment of planted native plants may highly depend on the density of the invasive exotic species [44]. However, it remains unclear how clipping alters the density effects of invasive plants on native plants.

Deepening understanding of the interactions between established invasive exotics and planted natives and combining these ecological principles with traditional approaches can be very promising in successfully controlling invasive exotics [13,16,17]. Although the effects of clipping invasive plants or planting native plants on restoration performance have been investigated in different systems [25,45], it remains unclear whether these two measures can work together to a better control efficiency. In this study, we explored the possibility of inhibiting the growth of an aggressively expanding exotic plant species, *Solidago canadensis* (Asteraceae), which is native to North America but now widespread in China, Europe, and other parts of the world [46,47,48,49]. This species is characterized with a large number of seed production (ca. 20,000 seeds produced per plant) and fast expansion with clonally rhizomatous growth, which lead to a very fast population increase rate [46]. Furthermore, this species possesses highly competitive capacities, repels natives, and forms large monocultural patches and causes substantial biodiversity loss [46,47,48,49]. Eradicating the aggressive *Solidago* plants is extremely difficult, often resulting in frequent re-invasion [32]. A study indicated that long-term herbicide spraying and mowing together with native seed addition can reduce *Solidago* coverage significantly [25]. However, the efficiency of this measure has to be examined in more studies. We asked the following questions: (1) Does clipping effectively inhibit the growth of invasive plants and promote the growth of competitive co-occurring species at different invasive plant densities? (2) Does planting the plants of the co-occurring species inhibit the growth of invasive plants in addition to clipping?

## 2. Materials and Methods

We examined the effects of aboveground clipping and sowing seeds of the two co-occurring and competitive plant species on biomass accumulation in invasive *S. canadensis* plants at five different densities using a randomized block design. The experiment was designed in a way simulating the circumstances in restoration of invaded habitats with two stages. In Stage I, the *S. canadensis* seedlings were cultivated at different densities, to simulate established *S. canadensis* populations with different densities in the field and to establish priority effects of *S. canadensis* against the planted co-occurring species in Stage II. In Stage II, the *S. canadensis* seedlings were subjected to different clipping treatments (clipping or non-clipping, setting up different priority effects) and treatments of competition with planted plants of co-occurring species (seed sowing of two co-occurring species, setting up different biotic resistance), to simulate the competition between established *S. canadensis* and planted plants in restoration projects.

### 2.1. Stage I: Culture of S. canadensis Seedlings at Different Densities

In November 2021, *S. canadensis* seeds were collected from eight populations in an old field (30°16′ N, 121°10′ E) in Ningbo City, an area where the invasion of *S. canadensis* is frequently observed. These seeds were stored at 4 °C until germinated in Petri dishes with moistened filter paper in a growth chamber (PPFD of 180 µmol·m^−^^2^·s^−^^1^, 24 °C during the day (12 h) and 18 °C at night (12 h)) on 7 April 2022. One week after germination, healthy and uniform seedlings with two cotyledons were transplanted into plastic pots in a partially climate-controlled greenhouse in the experimental garden of the Subtropical Forestry Institute at the Chinese Academy of Forestry. The seedlings were planted at five densities: one, two, three, four, and five seedlings per pot, with 36 pots for each density. The seedlings in each pot were planted at equal distances from each other using a circular design. Each pot was planted with at least two additional plants to protect against transplant mortality. After 1 week, appropriate test densities were established with thinning or the addition of new seedlings of the same age. These plants were cultivated until they were exposed to different treatments of clipping and sowing seeds of co-occurring species. Plastic pots (18 cm in diameter and 18 cm in height) were filled with approximately 4 L of a growth substrate. The substrates consisted of a completely mixed field-collected soil and organic matter (*v*/*v* = 4:1). Soil was collected from the upper 15 cm of the profile from an abandoned field near where *S. canadensis* seeds were collected. The pots were rearranged once per week to minimize the effects of environmental heterogeneity within the greenhouse. During the experiment period, light intensity inside the greenhouse was about 80% of the ambient light outside, with a daily maximum of 800–1200 µmol·m^−2^·s^−1^. Inside the greenhouse, the daily maximum air temperature fluctuated in the range of 25–38 °C during the day and the minimum value fluctuated in the range of 18–28 °C at night. The day length was 11–14 h. Soil moisture was monitored, and the plants were watered once or twice a day, depending on the air temperature. The soil contained 10.36 g kg^−^^1^ of organic matter (completely decomposed plant litters), 0.53 g kg^−^^1^ of total nitrogen, and 0.63 g kg^−^^1^ of total phosphorus and had a pH of 8.20.

### 2.2. Stage II: Clipping and Sowing Seeds of Co-Occurring Plant Species

An additive design competition experiment [50] was conducted to examine the effects of clipping and adding seeds of the co-occurring species to *S. canadensis*. Two common co-occurring plant species, *Imperata cylindrica* (Poaceae), a grass with dense ramets and rhizomes, and *Sesbania cannabina* (Leguminosae), an annual legume without clonal growth, were selected as the target species. These species were selected because they are the dominant plant species that typically form monocultural stands in abandoned fields and have application potentials in restoring *S. canadensis*-invaded habitats. Although *I. cylindrica* stands are often invaded by *S. canadensis*, *S. canadensis* can hardly invade the stands of *S. cannabina*, indicating the higher competitive ability of this species. The seeds of *I. cylindrica* were collected in June 2021 and the seeds of *S. cannabina* in November 2021 with the same methods described for collecting the seeds of *S. canadensis*. 

Stage I *S. canadensis* seedlings were exposed to a combination of three factors: (1) *S. canadensis* density (described in Stage I), (2) clipping or no clipping, and (3) sowing of *I. cylindrica* or *S. cannabina* seeds or no sowing. On 25–27 June 2022, half of the *S. canadensis* plants from each density treatment were clipped, and the other half were kept intact. For each half of these plants, five seeds of *I. cylindrica*, *S. cannabina*, or no seeds were sown in the center of the pots, with six replicates for each sowing treatment. Additionally, another five seeds were sown in each of the ten pots for each species without the presence of *S. canadensis* plants as a control. After 5 days, three to five seeds germinated, and the seedlings were thinned to one seedling after 1 week of growth. During the *S. canadensis* regrowth period, air temperature fluctuated between 24 and 38 °C/20 and 30 °C day/night conditions, and the plants were watered as described in Section 2.1.

On 15 September, 3 months after clipping and planting the plants from the two co-occurring species, all plants were harvested, the roots were carefully rinsed to remove the soil, and the roots of the two species grown in the same pot were separated. The leaves, stems, roots, and rhizomes from each plant were separated and dried at 80 °C for 6 days to constant mass and weighed.

### 2.3. Data Analysis 

The effects of density, clipping, and planting the plants from the two co-occurring species and their interactive effects on belowground biomass, aboveground biomass, and total biomass of *S. canadensis* were analyzed using a three-way ANOVA. Different *S. canadensis* density and clipping treatments were combined as a fixed factor, and a one-way ANOVA was conducted to determine their effect on the biomass of *I. cylindrica* and *S. cannabina*. All analyses were performed using fitting general linear models (GLM). Furthermore, the biomass of *S. canadensis* regressed against that of the paired *I. cylindrica* and *S. cannabina* grown in the same pot. When necessary, the data were transformed to meet the assumptions of normality and equal variance. The Fisher’s least significant difference (LSD) test was used to examine the differences among treatments at a 5% significance level. All analyses were performed using Statistical Product and Service Solution (SPSS) software (version 16.0; SPSS Inc., Chicago, IL, USA).

## 3. Results

### 3.1. The Effects of S. canadensis Density, Clipping, and Co-Occurring Species Planting on Biomass Accumulation of S. canadensis

**Figure 1:**
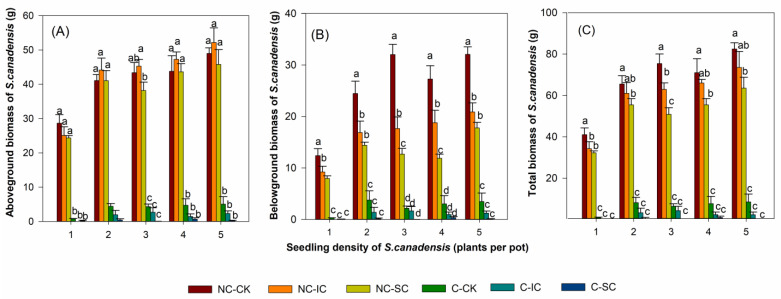
Biomass of the invasive *Solidago canadensis* seedlings exposed to different treatment of densities, clipping, and sowing seeds of the two co-occurring species. (**A**) Aboveground biomass; (**B**) belowground biomass; (**C**) total biomass. NC-CK, without clipping and seed sowing; NC-IC, without clipping and with *Imperata cylindrica* seed sowing; NC-IC, without clipping and with *Sesbania annabina* seed sowing; C-CK, clipping, without seed sowing; C-IC, with clipping and *I. cylindrica* seed sowing; C-SC, with clipping and *S. cannabina* seed sowing. The data are the means ± standard error and different letters indicate significant differences (*p* < 0.05) between the different treatments within each *S. canadensis* seedling density.

The treatments of clipping, sowing seeds of co-occurring species, and *S. canadensis* density had significant effects on the aboveground, belowground, and total biomass of *S. canadensis* (*p* < 0.05, Table 1). Clipping and seed sowing had significant interactive effects on belowground and total biomass (*p* < 0.05), but not on the aboveground biomass of *S. canadensis* (*p* > 0.05). There were significant interactive effects of clipping and density on the biomass of *S. canadensis* (*p* < 0.05). Significant interactive effects of *S. canadensis* density and seed sowing were observed only for belowground biomass (*p* < 0.05). No significant interactive effects of *S. canadensis* density, clipping, and seed sowing were observed (*p* > 0.05). The total biomass of *S. canadensis* per pot increased with *S. canadensis* density (Figure 1). Clipping decreased *S. canadensis* biomass significantly (*p* < 0.05), independent of *S. canadensis* density or seed sowing treatments. Planting the plants of the two co-occurring species significantly decreased the belowground and total biomass of *S. canadensis* in the non-clipping treatments (*p* < 0.05), but not in the clipping treatments, and *S. cannabina* seed sowing had greater effects than *I. cylindrica* seeds (Figure 1). The biomass of *S. canadensis* seedlings was reduced to a greater extent by clipping at higher densities than at lower densities compared with the corresponding non-clipped plants. 

### 3.2. The Effects of Clipping and Seedling Density on the Biomass of I. cylindrica and S. cannabina 

**Figure 2:**
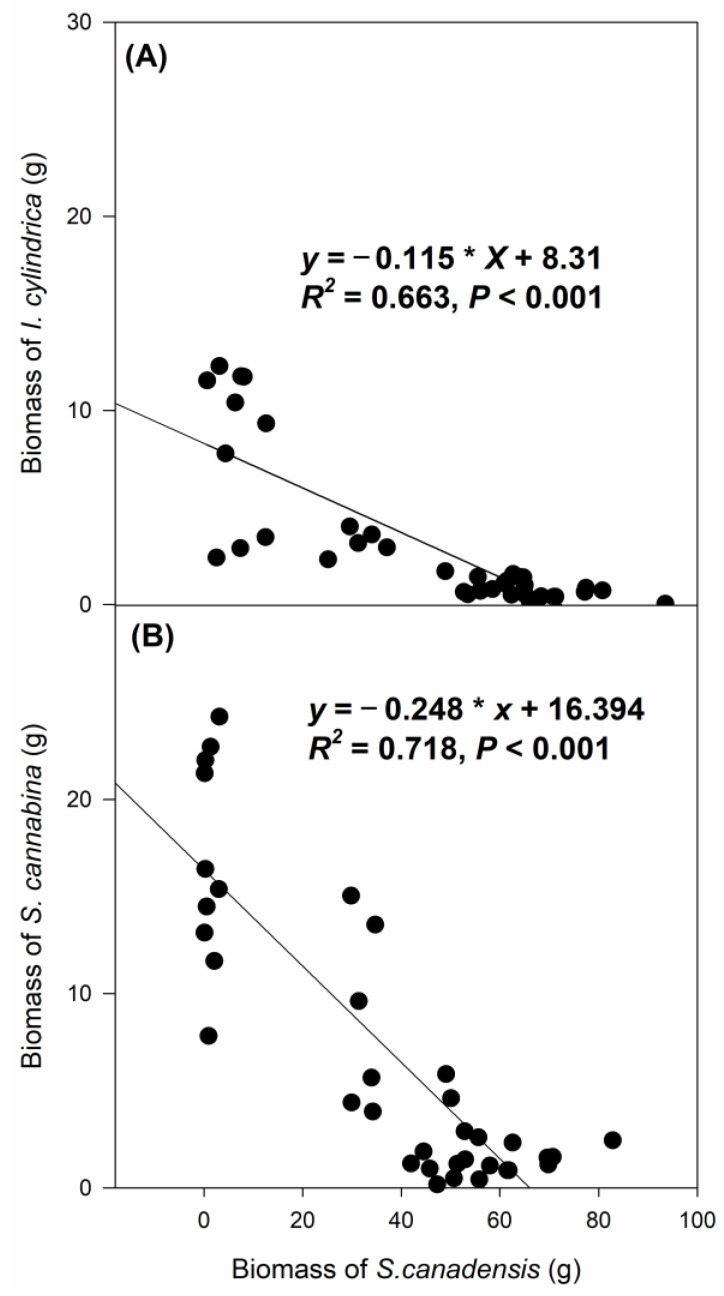
Regression of biomass of the plants of the co-occurring species with biomass of the *Solidago canadensis* seedlings. (**A**) *Imperata cylindrica* and (**B**) *Sesbania annabina*. The data were pooled from all the treatments in which *S. canadensis* was grown together with planted *I. cylindrica* or *S. cannabina*.

**Figure 3:**
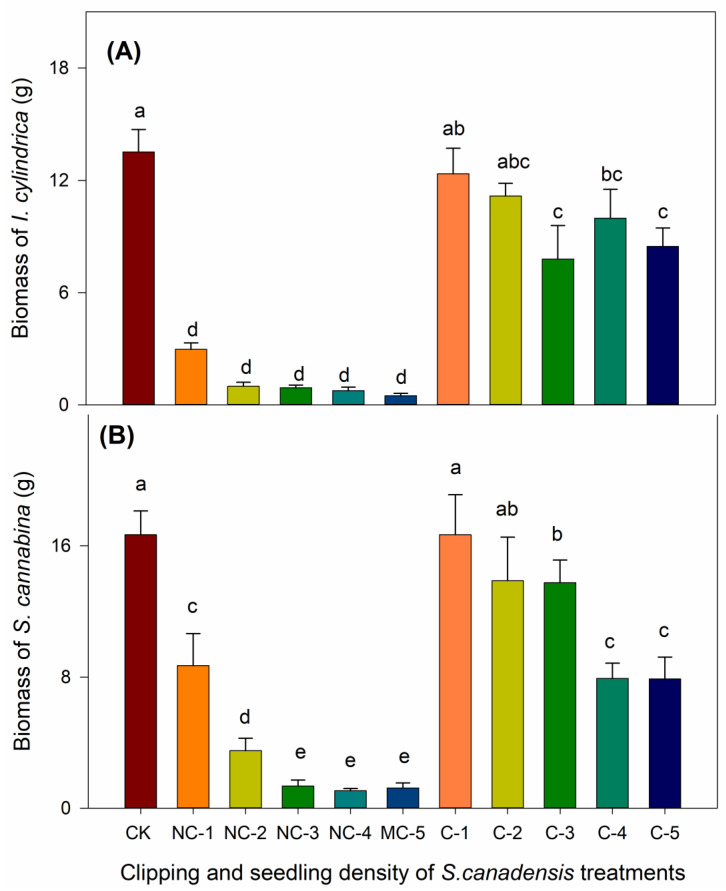
Total biomass of the seedlings of the co-occurring species exposed to different density and clipping treatments of the *Solidago canadensis* seedlings. (**A**) *Imperata cylindrica* and (**B**) *Sesbania cannabina*. CK, the *I. cylindrica* and *S. cannabina* seedlings were grown alone as a control (one seedling per pot). NC-1, NC-2, NC-3, NC-4, NC-5, and the *I. cylindrica* or *S. cannabina* seedlings were sown into the pots with 1–5 non-clipped seedlings of *S. canadensis* per pot, respectively. C-1, C-2, C-3, C-4, C-5, and the *I. cylindrica* or *S. cannabina* seedlings were sown into the pots with 1–5 clipped seedlings of *S. canadensis* per pot, respectively. The data are the means ± standard errors and different letters indicate significant differences (*p* < 0.05) between the different treatments within each *S. canadensis* seedling density for *I. cylindrica* and *S. cannabina*, respectively.

The combination of clipping and *S. canadensis* density significantly affected the biomass of *I. cylindrica* (D.f.10, 56, F = 23.746, *p* < 0.001) and *S. cannabina* (D.f.10, 53, F = 17.646, *p* < 0.001). *S. canadensis* suppressed the biomass accumulation of *I. cylindrica* and *S. cannabina* under all density treatments. The biomass of *I. cylindrica* and *S. cannabina* decreased significantly with increasing biomass of *S. canadensis* in the mixed culture treatments (*p* < 0.001; Figure 2 and Figure 3). The presence of non-clipped *S. canadensis* sharply decreased the biomass of *I. cylindrica* and *S. cannabina* (*p* < 0.05). In the non-clipping treatments, the biomass of *I. cylindrica* and *S. cannabina* decreased with the increasing density of *S. canadensis*, but this was only significant for *S. cannabina* (*p* < 0.05). Clipping significantly mitigated the suppression of *S. canadensis* biomass accumulation on *I. cylindrica* and *S. cannabina* in all *S. canadensis* density treatments (*p* < 0.05). In the clipping treatments, the biomass of *I. cylindrica* and *S. cannabina* growing with one or two *S. canadensis* seedlings was close to that of the corresponding monocultures (Figure 3). 

## 4. Discussion

The major purpose of this study is to investigate whether biotic resistance from planted plants of co-occurring species may further increase suppression effects on invasive exotics in addition to clipping. The experiment did find that planting the plants of co-occurring species suppressed the belowground growth of *S. canadensis,* although the effects were not significant when the plants were clipped. It suggested that combined treatments of clipping invasive exotic plants and plants of co-occurring species with high competitive abilities are promising in effectively controlling these aggressively expanding species. 

### 4.1. The Effects of Clipping and Planting Plants of the Co-Occurring Species on Biomass Accumulation of S. canadensis

Aboveground removal has long been commonly applied as the primary control measure of invasive exotic species [18,19,20]. In our experiment, the biomass of *S. canadensis* could be reduced by up to 90% by clipping, indicating the high effectiveness. These results are consistent with those of Knudson et al. (2012) and He et al. (2018), who observed that clipping effectively inhibited the growth of some invasive exotic species [41,45]. The efficiency of clipping in reducing plant growth highly depends on the regrowth potential, which may be affected by plant size and the pool size of nutrient reserves stored in belowground parts [51,52,53]. Larger plants have more nutrients stored underground that are ready to be remobilized to support a higher regrowth capacity [54]. In our experiment, *S. canadensis* seedlings were relatively small (two months old, in contrast to their long-lived perennial growth habit), which may explain their relatively high sensitivity to clipping and limited regrowth potential. 

The low efficiency, high cost, or environmental problems limit application of traditional measures, such as clipping, mowing, or fire and herbicide application, to control invasive exotic species [18,19,20]. Evidence indicates that restoring native cover can enhance invasion resistance [16,17,31,37]. We also observed significant effects of planting the co-occurring species on the growth of *S. canadensis*. Generally, the negative correlation between the biomass of *S. canadensis* and that of planted seedlings (Figure 2) indicated that there was strong competition between the planted seedlings of co-occurring species and the established *S. canadensis* plants. The seed sowing interfered with belowground growth but not aboveground growth of *S. canadensis*, suggesting intensive competition for root growth spaces, nutrients, or water. The findings comply with Szymura (2016), Ni (2018), and Ren (2019) [48,55,56], who found that roots play important roles in competition between native and invasive exotic species. The growth reduction regarding *S. canadensis* was significant in the non-clipping treatments but not in the clipping treatments. One explanation for the insignificant differences in the growth of clipped *S. canadensis* among the different sowing treatments is that the growth of these clipped plants is highly dependent on the number of dormant buds resprouting at the stem bases and the capacity to mobilize nutrient reserves in the rhizomes and coarse roots [46]. In contrast, in the non-clipping treatment, the major constraint for growth of *S. canadensis* may be the interference of planted seedlings of the co-occurring species. Despite the nonsignificant effects observed in the clipping treatments (Figure 1), regrowth of *S. canadensis* can be further reduced by planting seedlings of the co-occurring species, in addition to clipping. *S. canadensis* seemed to be more suppressed by *S. cannabina* than *I. cylindrica*, which was probably due to the larger plant size and faster biomass accumulation (Figure 3) of the former species, as competitive intensity is supposed to be dependent on neighbor biomass [57]. Interspecific variations in the suppression of exotic species using different native species have been observed [38,48], and selecting species with faster growth rates may enhance success in the initial stages of invaded ecosystem restoration [43,58]. Although *S. cannabina* is listed as an invasive species in China [59], it is a typical leguminous nitrogen-fixing species [60] and there is a long history of cultivation of *S*. *cannabina* as green manure to improve soil quality, especially that of saline or alkaline soil, by increasing soil organic matter and decreasing soil salinity and alkalinity [61,62], as forage for domesticated animals, and as a herbal medicine and a source of a food additive [63,64]. As *S*. *cannabina* serves as a multipurpose species in China currently, it can potentially be used in controlling *S*. *canadensis*. 

### 4.2. The Effects of S. canadensis on Biomass Accumulation of the Co-Occurring Species

Our experiment indicated that there were strong priority effects of *S. canadensis* over the co-occurring species, which have been observed in many other invasive exotics [39,65]. The two co-occurring species responded to the treatments of clipping and the density of *S. canadensis* similarly. However, the growth of *I. cylindrica* seemed to be more affected, probably because of the lower competitive capacity of this species [66]. These results suggest that there are variations in competitive tolerance between different co-occurring species and that screening co-occurring species with high competition tolerance may promote initial restoration success [58]. The mechanisms for the suppression effects of early established plants on later arriving plants may include shading from invasive plants [67,68], early pre-emption of essential resources in soil [69,70], suppression of native plants using allelopathic effects [71,72], and an altered soil biota community [73,74]. *S. canadensis* exhibited significant inhibiting effects on the growth of the two co-occurring species when they were not clipped or when clipped at a higher density, which offers an explanation for their dominance in the field [66,75]. Clipping significantly ameliorated the suppression effects, suggesting shading played an important role. But belowground parts of the invasive plants may also contribute to the suppression effects [27,35]. There were few significant differences between the aboveground biomass of clipped *S. canadensis* plants in the different density treatments (Figure 1), but biomass accumulation of *S. cannabina* and *I. cylindrica* was indeed lower in the higher *S. canadensis* density treatments, suggesting that there were stronger priority effects of the roots or rhizomes [27]. Denser roots and rhizome clumps may inhibit root growth and nutrient uptake in newly grown seedlings due to limited root growth space [27,35]. The results suggested that clipping alone may have very limited effects on the belowground priority effects. This may explain frequently observed fast regrowth of some invasive exotics and suppression of growth of planted natives in fields.

The findings from this study offer some implications for future studies and invaded habitat restoration. The results highlighted the importance of applying competitive native species and overcoming belowground priority effects of invasive exotic plants in restoration of native cover. Therefore, different components of the priority effects (belowground vs. aboveground; belowground components, such as nutrient competition, allelopathy, soil biota, etc.) and their contributions need to be identified, dissected, and quantified (13, 71) in future studies. Furthermore, interspecific variations in response to these components in different native plant species need to be clarified for optimized species selection. For restoration projects, spraying systemic herbicides with stronger effects on belowground parts, selecting native species with less sensitivity to the belowground priority effects of invasive exotics, and optimizing timing of control can be the most important components in future management plans [13,16,17,31].

## 5. Conclusions

The study indicated that in addition to clipping, planting co-occurring and competitive plant species can increase biotic resistance to further invasion of *S. canadensis* and both the aboveground part and belowground part of *S. canadensis* contributed to its suppression effects on planted co-occurring species. In the future, studies identifying the key components contributing to the priority effects of the established invasive exotic species over planted natives to optimize precise timing and selecting native species with high competitive capacities are extremely important for designing a successful restoration management.

## Figures and Tables

**Table 1:** The effects of clipping (C), sowing seeds of the two co-occurring species (S), and density of *S. canadensis* seedlings (D) on the biomass of *S. canadensis* seedling.

		Total Biomass		Aboveground Biomass		Belowground Biomass	
	D.f.	F	*p*	F	*p*	F	*p*
C	1	2094.975	**0.000**	2180.523	**0.000**	1134.113	**0.000**
S	2	25.144	**0.000**	5.678	**0.004**	72.507	**0.000**
D	4	31.018	**0.000**	26.545	**0.000**	25.248	**0.000**
C × S	2	5.224	**0.007**	1.873	0.158	34.010	**0.000**
C × D	4	20.753	**0.000**	17.601	**0.000**	16.271	**0.000**
S × D	8	0.840	0.569	0.419	0.908	2.661	**0.010**
C × S × D	8	0.478	0.870	0.413	0.912	1.991	0.052

C—*Solidago canadensis* clipping (clipping or non-clipping), S—seed sowing (without seed sowing, *Imperata cylindrica* seed sowing, or *Sesbania cannabina* seed sowing), D—density of *S. canadensis* seedlings (1, 2, 3, 4, or 5 plants per pot). The bold *p* value indicate significant effects.

## Data Availability

The datasets generated during and/or analyzed during the current study are available from the corresponding author upon reasonable request.
